# Major Complications Associated With Cerebrospinal Fluid Collection in 11 Dogs: Clinical Presentation and Imaging Characteristics

**DOI:** 10.1111/jvim.70165

**Published:** 2025-06-17

**Authors:** Cecilia‐Gabriella Danciu, Alana McCarthy, Abbe Crawford

**Affiliations:** ^1^ Veterinary Clinical Science and Services, Royal Veterinary College University of London Hatfield UK; ^2^ Small Animal Teaching Hospital University of Liverpool Liverpool UK

**Keywords:** canine, CSF, intracranial pressure, magnetic resonance imaging, mortality

## Abstract

**Background:**

Cerebrospinal fluid (CSF) collection is routinely performed in dogs with neurological disorders. Little is known about the rate of major complications associated with CSF collection.

**Objectives:**

Determine the number of dogs experiencing a major complication secondary to CSF collection in a single referral hospital.

**Animals:**

Eleven dogs.

**Methods:**

Single‐center, retrospective description of the number and nature of major complications encountered during or after CSF collection in dogs.

**Results:**

From 7545 CSF collections performed between 1998 and 2024, 11 dogs (0.15%) experienced a major complication. Eight of these dogs had abnormal mentation on presentation. The most common neuroanatomical localization was multifocal (5/11). Nine dogs underwent magnetic resonance imaging (MRI) of the head and one dog had radiographs of the cervical vertebral column before CSF collection. The most common MRI findings included effacement of the cerebral sulci (7/9) and dilatation of the ventricular system (5/9). Cerebrospinal fluid collection was performed in 11 dogs, of which 3 were unsuccessful. Analysis of CSF aided in the diagnosis of meningoencephalitis of unknown origin (3/8), cryptococcosis (1/8), lymphoma (1/8), and results were normal in one and non‐specific in two dogs. Eight dogs were euthanized after failure to recover spontaneous ventilation (6), cardiopulmonary arrest (1) or severe neurological deterioration (1). One dog died during cardiopulmonary resuscitation. Repeat MRI after CSF collection identified caudal transtentorial and foramen magnum herniation in one dog. Two dogs survived to hospital discharge but with permanent neurological deficits.

**Conclusion:**

The frequency of major complications following CSF collection was low, but the associated mortality was high. Abnormal mentation and MRI evidence of cerebral cortical swelling were commonly detected before CSF collection in dogs experiencing complications.

**Abbreviations**
CSF: cerebrospinal fluid
CNS: central nervous system
FLAIR: fluid attenuated inversion recovery
ICP: intracranial pressure
MRI: magnetic resonance imaging
MUO: meningoencephalitis of unknown origin
PDPH: post‐dural puncture back pain and headache
RI: reference interval
SRMA: steroid‐responsive meningitis‐arteritis
T2W: T2‐weighted
T1W: T1‐weighted
TNCC: total nucleated cell count
TP: total protein concentration

## 1 Introduction

Cerebrospinal fluid (CSF) is an ultrafiltrate of plasma that surrounds and permeates the entire central nervous system. It is produced by the choroid plexus, parenchymal and leptomeningeal capillaries and plays important roles in brain metabolism and regulation of intracranial pressure (ICP) [1, 2]. Cerebrospinal fluid fills the ventricular system and subarachnoid space, and communicates with the interstitial fluid of the central nervous system (CNS) [1, 3, 4]. Given its extensive distribution across the CNS, and the potential to collect it relatively non‐invasively, CSF analysis can provide valuable clinical information in the investigation of suspected non‐infectious inflammatory [5], infectious [6] and neoplastic diseases of the CNS [7].

In dogs, CSF typically is collected under general anesthesia using an aseptic technique by trained personnel, either from the cerebellomedullary cistern or the caudal lumbar subarachnoid space [8]. It generally is considered a relatively safe and non‐invasive procedure, but careful patient evaluation is required to identify any potential contraindications, such as increased ICP, coagulopathy, atlanto‐axial instability, cervical trauma, and local dermatitis at the site of collection [2, 8]. The decision to proceed with CSF collection is based on the evaluation of clinical and imaging variables, alongside careful consideration of the likely diagnostic yield and overall risk–benefit for the individual patient [9, 10, 11]. Minor complications associated with CSF collection have been reported in dogs and include the development of a subcutaneous hematoma, local dermatitis, or the inability to collect CSF [12]. More severe complications have been infrequently reported, including progressive myelomalacia [13, 14] and hematomyelia [15] after lumbar CSF collection in dogs. A retrospective study of 30 dogs with histopathologically confirmed intracranial neoplasia documented a complication secondary to CSF collection in 17% of the dogs, with the reported complications including coma, stupor and apnea [16]. Information on the rate of major complications arising as a result of CSF collection in dogs with other neurological disorders is needed. Additionally, potential risk factors for such complications, including neurological examination findings and magnetic resonance imaging (MRI) features, have not been characterized.

Our aim was to retrospectively determine the number of dogs experiencing a major complication secondary to CSF collection in a single referral hospital. Additionally, we aimed to describe any complications encountered, as well as the clinical presentation, imaging features, diagnosis, and outcome in the affected dogs.

## 2 Materials and Methods

Ours was a retrospective observational study from a single veterinary referral hospital, with ethical approval granted by the Social Science Research Ethical Review Board of the Royal Veterinary College (SR2019‐0533).

Digital medical records were searched from January 1998 until July 2024 for dogs in which (1) CSF collection was performed and (2) a major complication was recorded during or immediately after CSF collection with a detailed description of the event. A major complication was defined as an adverse event evident during or immediately after CSF collection requiring at least one of the following: (a) immediate therapeutic intervention, (b) prolonged hospitalization, (c) resulting in permanent neurological deficits, and (d) resulting in death or euthanasia of the dog. Cases were excluded if they had incomplete medical records or records were unavailable for review, and if the major complication was considered anesthesia or cardiac disease related. Dogs were assessed and had CSF collection performed by a board‐certified veterinary neurologist, neurology resident‐in‐training, or intern under the direct supervision of a board‐certified veterinary neurologist.

Information retrieved from the medical records of those dogs that experienced a major complication after CSF collection included signalment, presenting complaint, clinical and neurological examination findings, MRI findings, CSF collection site and results of CSF analysis (total nucleated cell count [TNCC], total protein concentration [TP] and cytology results). Where available, ancillary diagnostic tests, definitive diagnosis, repeat imaging findings, serial neurological examination findings, hospitalization time, treatment, and necropsy examination results were recorded. Follow‐up information was obtained from re‐examination appointments at the referral veterinary hospital or from the local veterinarian's medical records.

All MRI studies were performed using a 1.5 Tesla (T) device (Philips Intera, Philips Healthcare, Eindhoven, Netherlands). Dogs underwent general anesthesia and were positioned in sternal recumbency for MRI of the head. Anesthesia protocols varied among dogs, but MRI sequences included a minimum of transverse and sagittal T2‐weighted (T2W), transverse T2W fluid attenuated inversion recovery (FLAIR), gradient echo, T1‐weighted (T1W) pre‐ and post‐contrast images with additional T1W post‐contrast sagittal image after IV contrast agent administration (gadopentetate dimeglumine, 0.1 mmol/kg). Magnetic resonance images were assessed and evaluated by a board‐certified veterinary radiologist at the time of the diagnosis.

For the purposes of the study, MR images from all dogs that experienced a major complication during or immediately after CSF collection were re‐evaluated retrospectively using EUnity software by a board‐certified veterinary neurologist or neurology resident‐in‐training under the direct supervision of a board‐certified veterinary neurologist, and all abnormalities documented were described. Brain herniations were described based on previously published criteria [9, 11]. Changes in the internal and external CSF space were described, including effacement of cerebral sulci [11], ventricular compression, ventricular dilatation based on previous imaging criteria [17], compression of the interthalamic adhesion, and third ventricle flow artifact. The latter was based on T2W signal drop or lack of suppression of T2W*FLAIR images [18]. Changes in brain parenchyma were recorded including focal or diffuse increase in brain volume. Additionally, mass lesions and intracranial hemorrhage were described [19].

Commercially available software (IBM SPSS Statistics Desktop, Version 28.0, IBM Corporation, Armonk, New York) was used to report continuous variables. Continuous data then were tested for normality using the Shapiro–Wilk test, and non‐normally distributed data was reported as median and range.

## 3 Results

A total of 7545 dogs had CSF collected between January 1998 and July 2024. Of these, 11 dogs (0.15%) experienced a major complication. Three of the 11 dogs were cross‐breeds, with one Boston Terrier, Boxer, Cavachon, Cavalier King Charles Spaniel, French Bulldog, Irish Setter, Jack Russell Terrier, and Standard Schnauzer. There were five females, all neutered, and six males, of which four were neutered. The median age was 4 years (range, 5 months to 9 years). Clinical data are summarized in Appendix S1.

Presenting complaint and physical examination findings are summarized in Appendix S2. The most common neurological examination findings included obtundation (7/11), decreased to absent postural reactions (5/11), non‐ambulatory tetraparesis (4/11), and pathological nystagmus (3/11). Details of the abnormal findings of each dog's neurological examination are summarized in Appendix S1. Neuroanatomical localization based on the neurological examination findings was multifocal (5), brainstem (2), central vestibular system (2) and forebrain (1). One dog was neurologically normal with cervical hyperesthesia.

**FIGURE 1:**
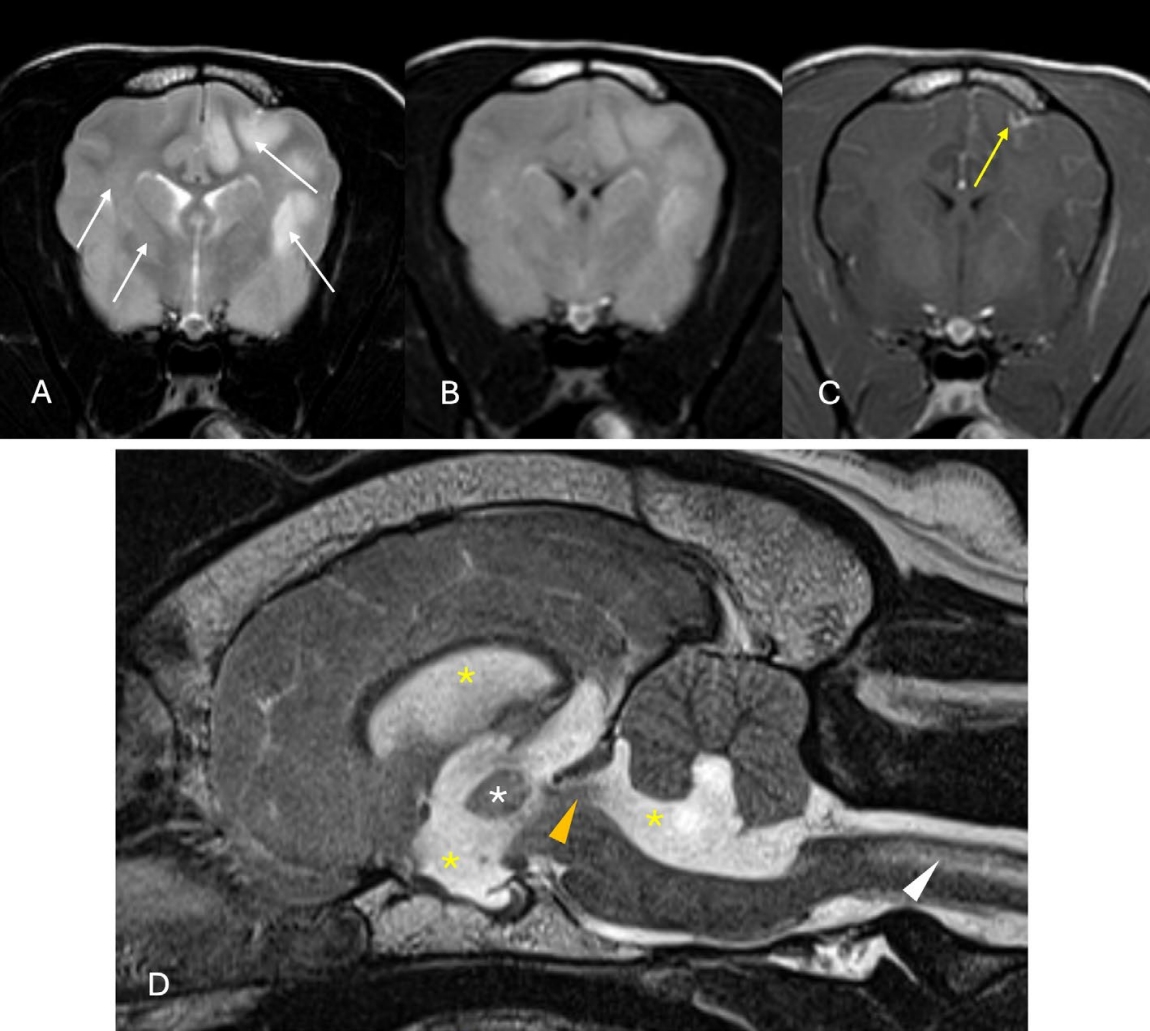
Magnetic resonance images of the brain of two dogs that had a major complication secondary to CSF collection (A–C: Dog 4, D: Dog 11). (A) Transverse T2‐weighted (T2W), (B) T2W fluid attenuated inversion recovery (FLAIR) and (C) T1‐weighted post‐contrast images at the level of the optic chiasm of Dog 4. There are multifocal, ill‐defined intra‐axial lesions (white arrows) in the gray and white matter which appear T2W and T2W‐FLAIR hyperintense, with focal contrast enhancement (yellow arrow). Additionally, there is generalized effacement of the cerebral sulci. (D) T2W sagittal view of the brain of Dog 11 shows generalized dilatation of the ventricular system (yellow asterisks). The interthalamic adhesion is reduced in size (white asterisk), and there is evidence of flow artifact in the mesencephalic aqueduct (orange arrowhead). There is moderate, poorly demarcated intramedullary T2W hyperintensity in the rostral aspect of the cervical spinal cord (white arrowhead). All changes are described relative to normal gray matter.

**FIGURE 2:**
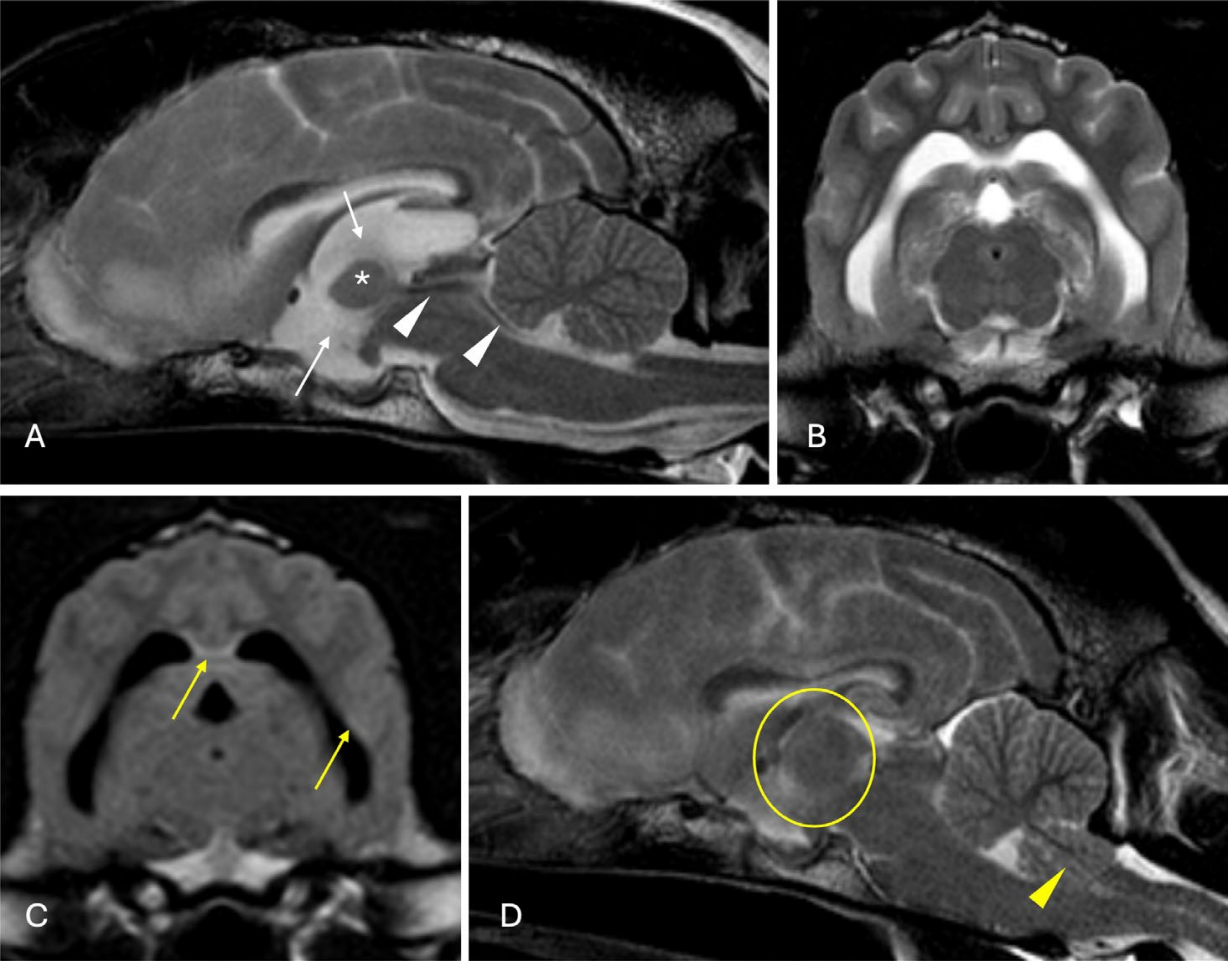
Magnetic resonance images of the brain of a 3.5 year old male neutered Standard Schnauzer (Dog 9) that encountered a major complication after CSF collection (failed to recover spontaneous ventilation). (A) T2‐weighted (T2W) sagittal image, (B) T2W and (C) T2W fluid attenuated inversion recovery (FLAIR) transverse images at the level of the midbrain. There is dilatation of the third ventricle (white arrows) with decreased of size and angulation of the interthalamic adhesion (asterisk) and T2W‐FLAIR periventricular hyperintensity (yellow arrows) compared to gray matter. Flow artifact is seen in the mesencephalic aqueduct and fourth ventricle (white arrowheads). (D) T2W sagittal image obtained post‐CSF collection: The third ventricle appears decreased in volume with expansion of the interthalamic adhesion (within the yellow circle) and foramen magnum herniation of the caudal aspect of the cerebellar vermis (yellow arrowhead). All lesions are described relative to normal gray matter.

Clinicopathological diagnostic test results are presented in Appendix S3. Diagnostic imaging was performed in 10 dogs before CSF collection, including MRI of the head (9) and vertebral column (4) and radiographs of the cervical and thoracic vertebral column in one dog. The radiographs were unremarkable. The most common findings on MRI of the head included effacement of the cerebral sulci (7/9), dilatation of the ventricular system (5/9), compression of the interthalamic adhesion (4/9), mesencephalic aqueduct flow artifact (4/9), meningeal contrast enhancement (4/9; Figure 1), and periventricular T2W/FLAIR hyperintensity (4/9; Figure 2C). Foramen magnum herniation was documented in 3/9 dogs, of which one was a Cavalier King Charles Spaniel with Chiari‐like malformation. Caudal transtentorial herniation was documented in 1/9 dogs. Individual MRI findings are described in Appendix S1 and summarized in Table 1.

**TABLE 1:** Summary of MRI findings in 11 dogs in which a major complication was documented during or immediately after CSF collection.

MRI variable	Number of dogs
Brain herniation	
Foramen magnum	3
Caudal transtentorial	1
Brain parenchymal changes	
Focal/diffuse increase in brain volume	3
Focal/multifocal mass lesion	2
Periventricular T2W/FLAIR hyperintensity	4
Diffuse/multifocal T2W/FLAIR hyperintensity	3
Cerebrospinal fluid space changes (internal and external)	
Effacement of the cerebral sulci	7
Compression of the ventricular system	3
Dilatation of the ventricular system	5
Expansion of the third ventricle	3
Compression of the interthalamic adhesion	4
Third ventricle/mesencephalic aqueduct flow artifact	4
Other	
Meningeal contrast enhancement	4
T2W hyperintensity within the cervical spinal cord	2

Abbreviations: CSF, cerebrospinal fluid; FLAIR, fluid attenuated inversion recovery; MRI, magnetic resonance imaging; T2W, T2‐weighted.

Cerebrospinal fluid collection was performed from the cerebellomedullary cistern in 6/11 dogs, from the lumbar subarachnoid space in 3/11 dogs, and the site was not recorded for 2/11 dogs. Collection was unsuccessful in a further three dogs. On attempting CSF collection in one dog (Dog 5), blood appeared in the needle hub, after which the dog deteriorated with hypoventilation, hypertension, and bradycardia, and additional attempts at collection were aborted. Collection was unsuccessful in the other two dogs despite multiple attempts (Dogs 2 and 6).

The results of CSF analysis from the eight dogs in which CSF collection was successful are reported in Appendix S4. The median TNCC was 390 cells/mm^3^ (range, 87–4023 cells/mm^3^). The median TP was 0.68 g/L (range, 0.27–0.80 g/L). Diagnosis was achieved with the aid of CSF cytologic analysis in five dogs (Dogs 3, 4, 9, 10, 11). Lymphocytic pleocytosis was documented in 3/5 dogs, of which one was diagnosed with granulomatous meningoencephalitis (confirmed on histopathology; Dog 3), one dog was diagnosed with presumed meningoencephalitis of unknown origin (MUO; Dog 4), and one with CNS lymphoma (Dog 10). In one dog, the cytology was characterized by mixed cell pleocytosis with cryptococcal organisms (Dog 9). One dog had mononuclear pleocytosis and was diagnosed with suspected MUO (Dog 11). In the remaining three dogs for which CSF analysis was performed, one sample was of insufficient volume to enable full analysis, but a mixed cell pleocytosis with neutrophilic predominance was reported on cytology (Dog 7), one had hemorrhagic contamination preventing analysis (Dog 1), and one had normal CSF (Dog 8).

Based on a combination of clinical findings, imaging features, CSF analysis, and where available, necropsy examination, a definitive diagnosis of granulomatous meningoencephalitis was made in two dogs (Dog 1 and 3); a territorial ischemic stroke in the region of the rostral cerebellar artery was diagnosed in one (Dog 6), cryptococcal meningoencephalitis in one (Dog 9), congenital hydrocephalus in one (Dog 2), and CNS lymphoma in one dog (Dog 10). A presumptive diagnosis of MUO was made in one dog (Dog 4), steroid‐responsive meningitis‐arteritis (SRMA, Dog 5) in one dog, and acquired hydrocephalus with possible MUO in one dog (Dog 11). A right olfactory bulb mass lesion was documented in one dog (Dog 7) at necropsy examination, but histopathology was not performed. No diagnosis was reached in one dog (Dog 8).

During or immediately after CSF collection, all dogs developed a major complication characterized by failure to recover spontaneous ventilation, cardiopulmonary arrest, marked deterioration in neurological status, or some combination of these. Two dogs survived to hospital discharge, 8 were euthanized, and 1 died.

Of the two dogs that survived to hospital discharge, one (Dog 2, congenital hydrocephalus) manifested new vestibular ataxia with a right‐sided head tilt on recovery from general anesthesia. Supportive care, including nursing, physiotherapy, and dexamethasone treatment (0.2 mg/kg IV single dose) was initiated before transitioning onto prednisolone (1.5 mg/kg PO q24h) and omeprazole (1 mg/kg PO q12h). This dog was discharged with mild right‐sided vestibular ataxia and right head tilt 3 days after CSF collection. In the other dog (Dog 5, suspected SRMA) blood appeared in the hub of the needle immediately after penetrating the subarachnoid space, which coincided with acute onset of hypoventilation, hypertension, and bradycardia. No further attempts at CSF collection were made. After recovery from general anesthesia, the dog had a generalized tonic clonic epileptic seizure, which was controlled with diazepam (0.5 mg/kg IV). Treatment with prednisolone (2 mg/kg PO q24h) and phenobarbital (2.5 mg/kg PO q12h) was initiated. The dog remained hospitalized and was noted to be blind in both eyes. Because of financial constraints, no further diagnostic tests were performed. This dog was discharged 6 days later, at which time it remained blind in both eyes.

Of the eight dogs that were euthanized, four failed to recover spontaneous ventilation after general anesthesia and, despite mechanical ventilation (for up to 12 h) with or without administration of mannitol and dexamethasone, no improvement was seen, prompting euthanasia (dogs 1, 3, 8 and 11). Three of these dogs were suspected or later confirmed on necropsy examination to have MUO (Dogs 1, 3 and 11), and one dog remained with an open diagnosis (Dog 8). Two dogs failed to recover spontaneous ventilation and additionally showed loss of brainstem reflexes (Dog 7 [diagnosed on necropsy examination with olfactory bulb neoplasia] and Dog 9 [diagnosed with cryptococcal meningoencephalitis]), which prompted euthanasia. Repeat MRI in Dog 9 identified caudal transtentorial and foramen magnum herniation, with brainstem compression and a generalized decrease in the volume of the ventricular system (Figure 2). One dog (Dog 10, diagnosed with CNS lymphoma) experienced cardiopulmonary arrest after CSF collection. The dog was successfully resuscitated, but the owner elected euthanasia without further treatment. One dog (Dog 6, diagnosed with right rostral territorial cerebellar artery ischemic stroke) was euthanized because of the severity of its neurological deterioration (comatose with absent brainstem reflexes). Finally, one dog (Dog 4, presumed diagnosis of MUO) died after cardiopulmonary arrest 10 h after general anesthesia. This dog had been dysphoric and severely obtunded immediately after general anesthesia (these deficits were not present before general anesthesia) and progressively deteriorated to cardiopulmonary arrest.

Necropsy examination was performed in three dogs. In Dog 7, in which CSF collection was performed without prior advanced imaging, the necropsy examination identified a large extra‐axial mass at the level of the right olfactory bulb causing a mass effect with secondary foramen magnum herniation. Histopathology was not performed in this dog. Changes consistent with granulomatous meningoencephalitis based on histopathological examination were documented in the other two dogs (Dogs 1 and 3).

Follow‐up information was available for the two dogs that survived to hospital discharge. Dog 2 underwent re‐examination 1 month after hospital discharge, and the vestibular ataxia had improved, but occasional leaning to the right persisted. The prednisolone dosage then was decreased to 0.25 mg/kg PO q24h. At the 2‐month re‐examination, mild paresis was documented in the right pelvic limb, but the vestibular ataxia had resolved. Dog 5 had a follow‐up evaluation 2 weeks after hospital discharge, at which time no additional seizures had been observed. The dog remained blind in the left eye, with an absent menace response and intact pupillary light reflex, but was visual in the right eye with an inconsistent menace response and intact pupillary light reflex. The prednisolone dose was decreased to 1 mg/kg PO q24h. At the 3‐month follow‐up, no seizures were reported, but the dog remained blind in the left eye.

## 4 Discussion

Our aim was to determine the frequency of major complications secondary to CSF collection in dogs at a single referral hospital. Over approximately 25 years, 0.15% of CSF collections resulted in a major complication, defined as an adverse event requiring immediate therapeutic intervention, or resulting in permanent neurological deficits, euthanasia, or death, or a combination of these. A recent observational multicenter study prospectively evaluated 102 dogs undergoing CSF collection, and no major complications were encountered [12]. Thus, although complications secondary to CSF collection appear rare, dogs deemed to have a clear contraindication would presumably not have undergone CSF collection, and hence these low risks occur in a pre‐selected population.

In people, the frequency of complications after CSF collection varies widely across studies (0.01%–30%) [20, 21, 22], with documented complications including subdural hematoma [20], post‐dural puncture back pain and headache [21, 22], arachnoiditis [23], and post‐dural puncture pseudomeningocele [24]. Although these specific complications were not definitively documented in our study population, only one dog underwent repeat MRI after CSF collection, and hence we do not know the specific cause of each dog's clinical deterioration. In Dog 5, blood filled the hub of the needle immediately on entering the subarachnoid space, and this occurrence coincided with marked clinical deterioration, including hypoventilation, hypertension, and bradycardia. This situation was suspected to reflect hemorrhage into the cerebellomedullary cistern with potential increase in ICP. This dog was provisionally diagnosed with SRMA, and hence a vasculitis in the cervical meninges might have predisposed to hemorrhage [25]. That being said, CSF collection is a standard diagnostic test in dogs with suspected SRMA, and hemorrhage resulting in postoperative neurological deterioration appears to be very rare. Advanced imaging of the vertebral column was not performed before CSF collection in this dog, nor after neurological deterioration, and hence the nature of the complication remains speculative. Collection of CSF was unsuccessful in two additional dogs, and it is possible that their deterioration was related to iatrogenic injury as a consequence of needle penetration of neural parenchyma, hemorrhage, or both.

In people, the most commonly documented complication is post‐dural puncture back pain and headache (PDPH) as a result of CSF leakage caused by the dural puncture [21, 22]. The precise pathomechanism of PDPH is not clear, but the tension caused by CSF leakage on pain‐sensitive meninges, meningeal vessels, and cranial nerves, aggravated by the orthostatic posture in humans, is hypothesized to be a key factor [26, 27]. This phenomenon is not yet recognized in dogs, likely because of their quadrupedal stance but also potentially because of the challenges of definitively identifying headache in dogs [28, 29].

Increased ICP is a contraindication to CSF collection because of the potential for neural structures to move from the high pressure intracranial compartment to a lower‐pressure spinal subarachnoid space, the pressure of which can be decreased further by CSF collection, resulting in herniation of brain parenchyma [2, 8, 30]. Herniation of the uvula of the cerebellum through the foramen magnum causes compression of the respiratory centers in the medulla oblongata [30]. Direct determination of ICP is invasive and rarely performed in clinical veterinary practice [31], and thus clinical findings and MRI features must be evaluated for potential indicators of increased ICP. Clinical signs can be relatively non‐specific such as obtundation, pain on palpation of the cranium, and proprioceptive ataxia [9, 10, 11, 32]. In the dogs in our study, the most common neurological abnormality identified was obtundation, which can reflect primary neurological dysfunction or systemic compromise [33]. That being said, obtundation was identified as an early negative prognostic indicator in dogs with MUO [34, 35], and can serve as an indicator of more severe inflammation or neuroparenchymal compromise. Interestingly, three dogs had normal mentation at the time of presentation, and recent literature suggests that increased intracranial pressure is not always clinically identifiable in dogs with neurological disease [9, 10]. Furthermore, Dog 2 was diagnosed with congenital hydrocephalus, a condition that typically has a more chronic, slowly progressive presentation and thus might be anticipated to have a lower risk of decompensation after CSF collection. Thus, advanced imaging is advised before collecting CSF to further evaluate for the presence and severity of increased ICP and remains prudent regardless of the underlying severity of presentation and diagnosis.

On MRI, mass effect, caudal transtentorial herniation, subfalcine herniation, perilesional edema, effacement of the cerebral sulci, and compression of the ventricular system have been documented in dogs with suspected increased ICP [9, 11]. However, reaching a diagnosis of increased ICP can be subjective, and determining which dogs are high risk remains challenging. The decision to collect CSF is further influenced by the risk–benefit ratio and the potential for the CSF analysis to influence subsequent management decisions. In our study, 9/11 dogs that experienced a major complication underwent MRI of the head and were deemed appropriate for subsequent CSF collection. However, herniation was documented in four dogs, and cortical swelling, visualized as effacement of the sulci or a mass lesion, was detected in seven dogs. Although non‐specific in terms of etiology, cortical swelling suggests increased parenchymal volume and hence potential for increased ICP [11]. Thus, the attending clinician presumably elected to proceed with CSF collection despite the potential for some degree of increase in ICP, deeming the potential benefits of collection and analysis to outweigh the risks of the procedure. This decision is not uncommon in clinical practice, where some degree of parenchymal pathology and swelling is common, and yet CSF collection proceeds without adverse effects. Specific criteria to identify which dogs are at higher risk of complications secondary to CSF collection would be valuable and warrant further investigation.

One dog in our study was diagnosed with cryptococcal meningitis. The MRI features of cryptococcosis include multifocal parenchymal disease with a predilection for gray matter, with additional meningitis, or meningitis with gelatinous pseudocyst formation and granulomatous mass lesions [36, 37]. Cerebrospinal fluid analysis is often a valuable tool in obtaining a diagnosis, with typical findings including a mixed or neutrophilic pleocytosis with direct visualization of cryptococcal organisms [36]. Despite prompt diagnosis and treatment, the prognosis in dogs with cryptococcosis is poor. In a previous study, 14% of dogs experienced cardiopulmonary arrest immediately after MRI and CSF collection [36]. Additionally, altered mental status decreased the chances of survival [36]. In the dog with cryptococcosis in our study, MRI documented a distended third ventricle, with compression of the interthalamic adhesion and flow artifact within the mesencephalic aqueduct. Repeat MRI after CSF collection disclosed collapse of the ventricular system with foramen magnum herniation, and hence the CSF collection is suspected to have resulted in a sudden change in ICP and subsequent herniation.

Interestingly, CSF flow artifact was found in 4/9 dogs in our study. Flow artifact appears as a decrease in normally high signal in T2W, with hyperintensity on T2W‐FLAIR images caused by failure of suppression of the CSF signal [18]. It can be caused by altered magnetization of the CSF during its movement close to the brainstem or within the mesencephalic aqueduct, also called entry slice phenomenon, and, on its own, is considered benign [18, 38]. However, it is more commonly seen where there is turbulent CSF flow, such as in hydrocephalus or syringomyelia [39]. Thus, flow artifact within the ventricles might indicate an increased risk during CSF collection in dogs and warrants further investigation as a potential MRI indicator of increased ICP.

Our study had some limitations, including those inherent in a retrospective study, such as variations in medical records and clinical management decisions for individual patients. Another important limitation is that it was not possible to definitively conclude that the clinical deterioration documented in each dog was a direct consequence of CSF collection. It is possible that some dogs deteriorated because of progression of their underlying disease. Severe or extensive pathology was documented on MRI in some of the included dogs, and it is possible that these dogs might have failed to recover from general anesthesia regardless of the CSF collection. Decompensation of attempts at autoregulation in dogs with increased ICP might have occurred during general anesthesia, with secondary foramen magnum herniation and respiratory compromise. Furthermore, some of the encountered complications could have been caused or exacerbated by an unrecognized adverse effect of the general anesthesia. Conversely, it is possible that we have underestimated the number of dogs experiencing a complication secondary to CSF collection because some might have gone unrecognized and presumed to be a result of disease progression. The precise cause of clinical deterioration after CSF collection remains speculative in 7/11 dogs because of the lack of follow‐up advanced imaging or necropsy examination. Additionally, the low number of dogs that experienced a complication during or immediately after CSF collection precluded meaningful statistical analysis, and hence our study is purely descriptive.

In conclusion, major complications related to CSF collection were documented in 0.15% of dogs undergoing CSF collection in our study population. Survival to discharge was low in dogs affected by a major complication (2/11). Obtundation and MRI evidence of cerebral sulci effacement were often present in dogs encountering a major complication following CSF collection. Additional research is needed to evaluate CSF collection‐related complications in a larger dog population across multiple veterinary hospitals and to develop specific clinical and MRI‐based guidelines to more accurately predict the risk of CSF collection for individual dogs.

## Disclosure

Authors declare no off‐label use of antimicrobials.

## Ethics Statement

Approved by the Royal Veterinary College Social Sciences Research Ethical Review Board (URN: SR2019‐0533). Authors declare human ethics approval was not needed.

## Conflicts of Interest

The authors declare no conflicts of interest.

## Supporting information


**APPENDIX S1.** Supporting Information.
