# Supplementary material for: Major Complications Associated With Cerebrospinal Fluid Collection in 11 Dogs: Clinical Presentation and Imaging Characteristics
Source: J Vet Intern Med. 2025 Jun 17;39(4):e70165. doi: 10.1111/jvim.70165 (PMC12172569; doi:10.1111/jvim.70165)
Supplement: Supplementary file 1 — APPENDIX S1. Supporting Information. [file JVIM-39-e70165-s001.docx]

Supplementary material

**S1.** Signalment, neurological examination, magnetic resonance imaging findings, diagnosis and outcome in 11 dogs encountering a major complication after cerebrospinal fluid collection.

| **Dog number** | **Signalment** | **Neurological examination findings** | **Neuroanatomical localization** | **Diagnosis imaging modality, and findings** | **Type of deterioration after the CSF collection** | **Type of intervention and outcome** | **Presumptive or confirmed diagnosis** |
| --- | --- | --- | --- | --- | --- | --- | --- |
| 1 | Cross breed, 2 year 2 months, MN, 17 kg | Stuporous, vertical nystagmus with absent menace response in both eyes, intact PLR in both eyes. | Brainstem | Brain MRI:  Diffuse loss of clear demarcation between grey and white matter. Generalized effacement of the cerebral sulci, reduction of the size of the third ventricle. Diffused marked enhancement of the leptomeninges in the parietal region. Caudal transtentorial and foramen magnum herniation. | Respiratory arrest immediately after CSF collection with failure to recover spontaneous ventilation. | Mechanical ventilation. Euthanasia due to failure to recover spontaneous ventilation. | Postmortem histopathology of the brain: granulomatous meningoencephalitis |
| 2 | Boston Terrier, 7 years, FN, 7.5 kg | QAR. Light palpation of the cranial thoracic vertebral column elicited phantom scratching. | Forebrain based on the history of a seizure-like event | Brain and cervical vertebral column MRI:  Generalized dilatation of the ventricular system, consistent with hydrocephalus with no evidence of obstruction. Caudal transtentorial herniation. Diffuse marked cervical syringomyelia. | New onset vestibular ataxia and head tilt following CSF collection. | Supportive nursing care, dexamethasone (0.2 mg/kg IV single dose), then 1.5 mg/kg oral prednisolone SID. Resolved over 2 months. | Congenital hydrocephalus and cervical syringomyelia |
| 3 | Jack Russel Terrier, 4 years, FN, 7.65 kg | Obtunded. Non-ambulatory tetraparetic with absent postural reactions in all four limbs, absent withdrawal reflex in the thoracic limbs. | Multifocal (brainstem and C6-T2 spinal cord segments) | Brain and cervical vertebral column MRI:  Ill-defined patchy intra-axial lesions within the forebrain, thalamus, midbrain, brainstem and cervical spinal cord extending from C2 to C6. All lesions appear T2W hyperintense compared to grey matter. Contrast enhancement present at the right corona radiata of the parietal area. | Failure to recover spontaneous ventilation after general anesthesia. | Mannitol (1g/kg IV), dexamethasone (0.2 mg/kg IV).  Mechanical ventilation for 45 minutes. Euthanasia. | Postmortem histopathology of the brain: granulomatous meningoencephalitis |
| 4 | Cavachon, 5 years, FN, 8 kg | Obtunded. Absent menace response in both eyes. Absent nasal septal response on the right. Absent paw replacement in the right thoracic and pelvic limbs. Weakly ambulatory with moderate general proprioceptive ataxia in all limbs. | Multifocal (left forebrain and brainstem) | Brain MRI:  Multifocal ill-defined lesions confined in the grey and white matter which appear T2W and FLAIR hyperintense in the area of the left parieto-temporal cortex, bilateral thalamus. Adjacent leptomeningeal contrast enhancement. Effacement of the cerebral sulci. | Dysphoria and severe obtundation after anesthesia. Progressive deterioration in neurological state with cardiopulmonary arrest after 10 hours. | Dexamethasone (2 mg/kg IV single dose) and cardiopulmonary resuscitation. Died despite attempt to resuscitate. | Presumed MUO |
| 5 | Cross breed, 10 months, ME, 8.1 kg | Low head carriage. Painful on palpation of the cervical and thoracolumbar vertebral column. | Neurologically normal with cervical and thoracic hyperesthesia | Lateral radiographs of the cervical and thoracic vertebral column were normal. | Hypoventilation, hypertension and bradycardia, generalized epileptic seizure. Blind in both eyes post CSF collection. | Diazepam (0.5mg/kg IV single dose), prednisolone (2mg/kg PO SID), phenobarbital (2.5mg/kg PO BID). Remained blind in the left eye at 3 months follow-up. | Suspected steroid-responsive meningitis-arteritis |
| 6 | Cavalier King Charles Spaniel, 8 years 3 months, FN, 7.8 kg | Left-sided head tilt, marked vestibular ataxia, right sided postural reaction deficits, subjectively reduced menace response in both eyes. Rotatory positional nystagmus with the fast phase to the left. | Central vestibular system (paradoxical) | Brain MRI:  Sharply demarcated, wedge-shaped T2W and FLAIR hyperintense and T1W hypointense lesion in the right cerebellar hemisphere. Mild mass effect, no contrast enhancement. Foramen magnum herniation of the uvula of the cerebellum. Chiari-like malformation with impingement of the ventral aspect of the occipital bone into the caudal cerebellum, rostrotentorial overcrowding, medullary kinking and syringomyelia in the visible portion of the cervical spinal cord. | Comatose with absent brainstem reflexes following discontinuation of general anesthesia. | Mechanical ventilation. Mannitol and dexamethasone (unknown dose). No improvement over 4 hours. Euthanasia. | Territorial ischemic stroke in the region of the right rostral cerebellar artery. Chiari-like malformation and syringomyelia. |
| 7 | Boxer, 3 years 5 months, MN, 29kg | Obtunded. Left head tilt. Non-ambulatory tetraparetic with absent postural reactions in all four limbs. Reduced menace response in the left eye with reduced facial sensation. | Multifocal (brainstem and C1-C5 spinal cord segments) | No advanced imaging performed. | Comatose with absent brainstem reflexes following discontinuation of general anesthesia. Did not recover spontaneous ventilation. | Mechanical ventilation. Mannitol 2 g/kg IV, methylprednisolone 2 mg/kg IV, furosemide 2 mg/kg IV. Progressed to bilateral miosis 4 hours after CSF collection. Euthanasia. | Necropsy: Suspected right olfactory bulb neoplasia. On gross examination there was a right olfactory bulb mass causing mass effect and cerebellar vermis herniation through the foramen magnum. |
| 8 | Irish Setter, 5 months, ME, 14 kg | Obtunded. Cerebellar ataxia with reduced postural reactions in all four limbs. Absent menace response and PLR in both eyes. Spontaneous vertical conjugate nystagmus in both eyes. | Multifocal (brainstem and cerebellum) | Brain MRI: Increased T2W signal of the cerebral sulci, which could represent reduced brain volume or brain atrophy. No other structural changes were noted. | Failed to recover spontaneous ventilation following discontinuation of general anesthesia. | Mechanical ventilation for 12 hours. Euthanasia. | Open diagnosis. |
| 9 | Standard Schnauzer, 3 years 5 months, MN, 17.3 kg | Obtunded. Generalized proprioceptive ataxia. Mild cervical hyperesthesia. | Brainstem, without excluding C1-C5 spinal cord segments | Brain and cervical vertebral column MRI:  Rounding and moderate dilatation of the lateral and third ventricles with fluid isointense to CSF. Periventricular T2W and FLAIR hyperintensity. The interthalamic adhesion is smaller and angular. Well-defined hypointense flow artefact within the mesencephalic aqueduct and rostral fourth ventricle. Cervical region normal. | Failed to recover spontaneous ventilation and remained comatose with absent brainstem reflexes after discontinuation of general anesthesia. Repeat MRI of the head was performed, showing caudal transtentorial and foramen magnum herniation, with brainstem compression and a generalized reduction in volume of the ventricular system. | Mechanical ventilation. Euthanasia. | Cryptococcal meningoencephalitis |
| 10 | Cross breed, 9 years, FN, 5.3 kg | Obtunded. Ventroflexion of the neck and episodic right head turn. Non-ambulatory tetraparesis. Decreased hopping in thoracic limbs. Episodic spontaneous jerk conjugate nystagmus with the fast phase to the right. | Multifocal (brainstem, C1-C5 spinal cord segments, with possible involvement of the forebrain) | Brain MRI:  Enlargement of the caudal part of the cerebellum with loss of arborization causing compression of the medulla oblongata. This area appears T2W hyper- and T1W hypointense compared to grey matter. Adjacent meningeal contrast enhancement is present. Focal ill-defined T2W and FLAIR hyperintense lesion in the grey matter of left cingulate gyrus. Bilateral symmetrical enlargement of the lateral ventricles. Foramen magnum herniation. | Developed bradycardia and cardiopulmonary arrest immediately after CSF collection. | Cardiopulmonary resuscitation with return of spontaneous breathing and heartbeat. Hypertonic saline 3ml/kg IV and dexamethasone 0.15 mg/kg IV. Euthanasia 10 hours after resuscitation. | Cerebellar mass lesion (CNS lymphoma based on CSF analysis) with secondary dilatation of the ventricular system and compression of the medulla oblongata. |
| 11 | French Bulldog, 9 years, MN, 17 kg | Obtunded. Right head tilt. | Central vestibular system (right sided) | Brain and cervical vertebral column MRI:  Enlargement of the ventricular system with fluid isointense to CSF, and T2W and FLAIR periventricular hyperintensity. The fourth ventricle distension is causing marked dorsal displacement of the cerebellum and ventral displacement with flattening of the dorsal margin of medulla oblongata. There is generalized effacement of the cerebral sulci. The interthalamic adhesion is reduced in size and angulated, and there is evidence of flow artefact in the mesencephalic aqueduct. Moderate intramedullary T2W hyperintensity in the entire cervical spinal cord. | Failed to recover spontaneous ventilation following discontinuation of general anesthesia. | Dexamethasone 0.15 mg/kg IV and mechanical ventilation maintained for 3 hours. Euthanasia. | Suspected acquired generalized hydrocephalus, presumed MUO. |

Abbreviations: CSF, cerebrospinal fluid; CNS, central nervous system; FN, female neutered; FLAIR, fluid attenuated inversion recovery; ME, male entire; MN, male neutered; MRI, magnetic resonance image; MUO, meningoencephalitis of unknown origin; PLR, pupillary light reflex; RI, reference interval; T1W, T1-weighted; T2W, T2-weighted; QAR, quiet alert responsive.

**S2.** Presenting complaint and physical examination findings of the 11 dogs that encountered a major complication after cerebrospinal fluid collection.

| Dog number | Presenting complaint | Physical examination findings |
| --- | --- | --- |
| 1 | 24-hour history of hyporexia, vomiting, pyrexia (39.6ºC/103.28ºF), and retropharyngeal swelling | Tachypnea, inspiratory stridor. Rectal temperature of 40.2ºC/104.36ºF |
| 2 | Three-day history of lethargy, and one epileptic seizure-like event. Previously diagnosed with congenital hydrocephalus and cervical syringomyelia | NAD |
| 3 | Two-week history of lethargy, which progressed to tetraparesis | NAD |
| 4 | Acute onset generalized and focal epileptic seizure activity | NAD |
| 5 | Four-day history of lethargy, hyporexia, pyrexia (39.8ºC/103.64ºF) and neck pain | NAD |
| 6 | Acute onset recumbency, shivering, hypersalivation | NAD |
| 7 | Two episodes of ataxia over two weeks, which progressed to lethargy | NAD |
| 8 | Cluster epileptic seizures, pyrexia and behavioral changes including aggression over 10 days | NAD |
| 9 | Four-week history of lethargy, reluctance to move. Abnormal episodes of stiffness of all limbs. | NAD |
| 10 | Two-day history of obtundation which progressed to non-ambulatory tetraparesis with episodes of neck and limb rigidity | NAD |
| 11 | Lethargy over 3-4 weeks, low head carriage, yelping when picked up | NAD |

Abbreviations: NAD, no abnormalities detected.

**S3.** Results of routine blood work and additional diagnostic tests of the 11 dogs that encountered a major complication after cerebrospinal fluid collection.

| Dog number | Routine blood work | Additional diagnostic tests |
| --- | --- | --- |
| 1 | Severe alkalemia (pH 7.737, RI 7.360-7.470) with a respiratory alkalosis (pCO2 9.6 mmHg, RI: 26.0-41.0), normal hematology and serum biochemistry | Partial thromboplastin time, activated thromboplastin time, buccal mucosal bleeding time, Baermann fecal analysis, thoracic radiographs and abdominal ultrasound – all normal. Necropsy examination. |
| 2 | NAD | Non-invasive blood pressure measurement, thyroxine and urine analysis - all normal. |
| 3 | NAD | Necropsy examination. |
| 4 | Normal hematology. Serum biochemistry: mild hypercholesterolemia (8.5 mmol/L; RI 3.2-6.2) and hypocalcemia (2.15 mmol/L; RI 2.18-2.70), rest within reference intervals. | - |
| 5 | Normal hematology. Serum biochemistry: mild hypocalcemia (1.06 mmol/L, RI 1.13-1.33 mmol/L), rest within reference intervals. | - |
| 6 | Normal hematology. Serum biochemistry: elevation of CK concentration at 908 U/L (RI 61-394), rest within reference intervals. | - |
| 7 | NAD | Toxoplasma and Neospora serology negative. Necropsy examination. |
| 8 | Normal hematology. Serum biochemistry: elevated CK concentration at 876 U/L (RI 61-394), rest within reference intervals. | Bile acid stimulation test, ammonia, thoracic radiographs and abdominal ultrasound – all normal. Negative for Distemper, Toxoplasma and Neospora serology. |
| 9 | Normal hematology. Serum biochemistry: elevated CK concentration at 712 U/L (RI 61-394), ALP 154 U/L (RI 0 – 130), rest within reference intervals. | Non-invasive blood pressure measurements – normal. |
| 10 | Normal hematology. Serum biochemistry: elevated creatinine concentration at 2.55 mg/dL (RI 0.5-1.7), rest within normal limits. | Non-invasive blood pressure measurements – normal. |
| 11 | NAD | - |

Abbreviations: ALP, alkaline phosphatase; CK, creatin kinase; NAD, no abnormalities detected.

**S4.** Cerebrospinal fluid analysis results in 11 dogs in which a major complication was documented during or immediately following CSF collection.

| Dog number | CSF collection site | TNCC (RI < 5 cells/mm^3^) | RBC (RI < 0 cell/mm^3^) | TP (<0.25 g/L cerebellomedullary cisternal sample, <0.45 g/L lumbar sample) | CSF cytology | Diagnosis |
| --- | --- | --- | --- | --- | --- | --- |
| 1 | Lumbar | 600 | 310000 | 0.70 | Mixed cell pleocytosis with hemorrhage | Hemorrhagic. contamination |
| 2 | Cerebellomedullary cistern – attempted but not successful | - | - | - | - | - |
| 3 | Lumbar | 2690 | 8 | 0.80 | Lymphocytic pleocytosis | Suspected MUO |
| 4 | Cerebellomedullary cistern | 370 | 0 | 0.56 | Lymphocytic pleocytosis | Suspected MUO |
| 5 | Cerebellomedullary cistern – blood appeared in the hub of the needle; no CSF collected | - | - | - | - | - |
| 6 | Lumbar – attempted but not successful | - | - | - | - | - |
| 7 | Not recorded | 87 | 5 | Insufficient sample | Mixed cell pleocytosis with neutrophil predominance | - |
| 8 | Not recorded | 0 | 0 | 0.19 | Normal | No diagnosis |
| 9 | Cerebellomedullary cistern | 390 | 90 | Insufficient sample | Mixed cell pleocytosis with Cryptococcal organism present | Cryptococcal meningoencephalitis |
| 10 | Cerebellomedullary cistern | 379 | 31 | Not performed | Lymphocytic pleocytosis with large lymphocytes containing round to indented nucleus. Low numbers of mitosis. | CNS lymphoma |
| 11 | Cerebellomedullary cistern | 4023 | 16875 | 0.27 | Mononuclear pleocytosis | Suspected MUO |

Abbreviations: CSF, cerebrospinal fluid; CNS, central nervous system; MUO, meningoencephalitis of unknown origin; TNCC, total nucleated cell count; TP, total protein; RBC, red blood cells; RI, reference interval.
